# Evaluating the immediate impacts of the repeal of the Northern Territory minimum unit price on off‐premises alcohol pricing: Findings from an observational study of online alcohol retailers

**DOI:** 10.1111/add.70466

**Published:** 2026-06-16

**Authors:** Mia Miller, Nicola Man, Nicholas Taylor, Michael Livingston, Sarah Clifford, Michala Kowalski, Heng Jiang, Sarah Callinan, Cassandra J. C. Wright, Amy Peacock

**Affiliations:** ^1^ National Drug and Alcohol Research Centre University of New South Wales Sydney Australia; ^2^ Menzies School of Health Research Charles Darwin University Darwin Australia; ^3^ National Drug Research Institute Curtin University Melbourne Australia; ^4^ Drug Policy Modelling Program University of New South Wales Sydney Australia; ^5^ Centre for Alcohol Policy Research La Trobe University Melbourne Australia

**Keywords:** alcohol, alcohol policy, alcohol price, minimum unit pricing, Northern Territory, policy evaluation

## Abstract

**Background and aims:**

A minimum unit price (MUP) of AUD$1.30 per standard drink was implemented in 2018 in the Northern Territory (NT), Australia, to reduce the availability of cheap alcohol. Despite evidence of its efficacy in decreasing alcohol consumption and harms, the MUP was repealed on 1 March 2025. This study aimed to determine the immediate impacts of the MUP repeal on the price of off‐premises (i.e. takeaway) alcohol in Darwin, NT, assessing the proportion of products available <$1.30 per standard drink after the repeal in March 2025, identifying whether these products were pre‐existing or new to market, and analysing any broader shifts in the price distribution across all alcoholic products that could be attributable to the repeal.

**Design and participants:**

Observational study of all alcoholic products available from three large alcohol retailer websites.

**Setting:**

Darwin, NT, between October 2024 and March 2025.

**Measurements:**

Price per standard drink was estimated, and fixed‐effects panel quantile regression was used to test for price differences.

**Findings:**

Within one month of the repeal, no beer or spirits, 5.1% of all wine and 2.5% of all ciders were available <$1.30 per standard drink, including 67.6% of the 37 wine products in vessels ≥1 L. Of the products identified at <$1.30 per standard drink, most had been available in previous months (i.e. not new or restocked). Quantile regression results showed a downward shift in price across the market for all product types, including beer and spirits, which were priced substantially above the $1.30 threshold at all percentiles. The largest reductions were observed for cheaper spirits, beer, cider and wine ≥1 L, ranging from $0.08 at the 20th percentile for spirits to $0.48 for wine ≥1 L at the 10th percentile.

**Conclusions:**

The minimum unit price repeal in Northern Territory, Australia, in March 2025 resulted in an immediate increase in the availability of cheap alcohol products and a downward shift in the overall price of alcohol in the off‐premises market, particularly for lower‐priced products.

## INTRODUCTION

Interventions that increase the price of alcohol are among the most effective and cost‐effective for reducing alcohol consumption and associated harm [1]. Minimum unit price (MUP) policies, which set a retail floor price below which alcohol cannot be sold, are one such approach recommended by the World Health Organization (WHO) [1]. As MUP targets the cheapest alcohol products, it is suggested to have the most substantial benefits for heavy drinkers as the group who primarily consume low‐priced alcohol [2], while concurrently shielding moderate drinkers from price increases [3].

The Northern Territory of Australia (NT) is one of the few jurisdictions globally to have implemented MUP, alongside Armenia, Scotland, Ireland, Wales and some Canadian provinces [4]. Introduced in October 2018, the NT MUP was set at AUD$1.30 per standard drink (10 g ethanol), with the objective of reducing the consumption of cheap, high alcohol content cask and bottled wine, without impacting the beer market [5]. The MUP had several different impacts on the price of off‐premises alcohol in the NT. Research undertaken in the year after implementation demonstrated that there were substantial reductions in the proportion of products sold below $1.30 (from 19% pre‐MUP to 1% post‐implementation), either because retailers complied with the policy or because cheap products were delisted [6]. Additionally, price rebalancing occurred in the market following MUP introduction, with some products that were priced well above the $1.30 per standard drink threshold also increasing in price [6]. This phenomenon has been observed in other jurisdictions when a minimum price policy has been implemented or the price threshold increased, including in Scotland and British Columbia, Canada [7, 8].

Evaluations of the NT MUP found that it was effective overall in reducing harms without negatively impacting moderate alcohol consumers or economic operators. In the 12 months following implementation, per capita cask wine consumption reduced by 50.6%, with no evidence of substitution to other alcoholic beverage types [5]. Reductions in alcohol‐related assault offences and alcohol‐related ambulance attendances were also observed [9, 10]. Simultaneously, the MUP had a negligible impact on moderate drinkers [11] and no adverse economic effects on alcohol retailers [12] or the tourism sector [10]. This evidence is largely in line with findings from other jurisdictions with MUP in place, which have seen decreases in alcohol sales [13, 14], alcohol‐attributable hospitalisations [15] and alcohol‐attributable deaths [16], with no corresponding, large‐scale negative social outcomes [17, 18] or impacts on industry revenue [18].

Despite evidence of its effectiveness, the MUP was removed on 1 March 2025 after the NT's conservative party won the August 2024 election on a platform that included the repeal [19]. The NT now represents the first instance of MUP being repealed globally. Given this lack of precedent, the extent of the impact the repeal may have on the alcohol market is currently unknown. This includes whether alcohol companies will reduce the price of alcohol, both of cheap wine and other products priced well above the MUP threshold, reintroduce products previously removed from the market on MUP implementation and/or introduce new, low‐cost products to the market. Understanding the impact of the repeal is crucial to inform future discussions on MUP, and pricing policies more broadly, given the challenges of implementing [20] (and now sustaining) these approaches. Determining the immediate impacts is particularly imperative in the context of the NT, which has the highest rates of alcohol‐related harm in Australia [21].

This article aims to provide early insights into any immediate impacts (i.e. within the first month) of the repeal of the MUP on the retail price of off‐premises alcohol in the NT, focusing on the capital city, Darwin. Specifically, by comparing the price of alcohol in March 2025 after the MUP repeal to alcohol prices in preceding months when MUP was in place, we aimed to:

1. Determine the proportion of products after the repeal that were priced under the previous $1.30 per standard drink threshold;
2. Determine the proportion of products priced under $1.30 after the repeal that were not previously available in the NT market pre‐repeal; and
3. Investigate whether the overall price distribution of products shifted after the repeal.

## METHODS

Data were collected from the websites of three major Australian alcohol retailers, with the location set to Darwin, the capital city of the NT. Unlike in most other Western countries, Australian sales data that include information on the price of alcohol at the product and brand level are unavailable for research purposes. As such, the data source used in this article represents to date the only publicly available, collated data on alcohol pricing in the NT.

One liquor store owned by each of the three retailers was selected for data collection. Retailer one had only a single store within the Darwin city postcode area (0800). For retailers two and three, the nearest liquor store to the retailer one store (defined by the mean of the straight‐line distance) was then selected. The size of alternative stores for these two retailers in Darwin (based on number of available products) was similar to the chosen store. Data from each website for every listed product were collected (set to that store location) during the last week of every month from October 2024 until March 2025. The selected store for the smallest retailer (retailer 2) closed between the December 2024 and January 2025 data collection; an alternative store from this retailer was selected for data collection from January 2025 until March 2025. All prices in this study are in Australian dollars (1AUD ≈ 0.65USD).

### Data collection and extraction

A list of uniform resource locators (URLs) for each product under the categories of spirits, premix, beer, cider and wine were obtained from each retailer website. Data were extracted from these URLs using semi‐automated Python scripts that were developed within our research team. Some retailers listed prices separately for purchase via delivery versus collection in‐store; we focused only on products available for collection in‐store.

Products in different vessel types (e.g. bottles versus cans) and sizes (e.g. 400 mL bottle versus 700 mL bottle) within a retailer, or the same product from a different retailer, were considered different products. The data from the product details page were transformed to yield multiple price observations where available (e.g. for different pack sizes).

#### Product identifier

Each unique beverage in a different pack size, vessel type and/or vessel size within each retailer was assigned a unique product identifier. In other words, the same beverage in different pack sizes (e.g. a pack of 6 × 355 mL bottles versus a single 355 mL bottle), vessel types (e.g. a bottle versus a can) and/or vessel sizes (e.g. a 400 mL bottle versus a 700 mL bottle) within a retailer, or the same beverage from a different retailer, were considered different products. The product identifier identified the unique product over time in this study.

#### Product type

Alcoholic beverages were categorised into four product types, using the algorithm described in the Supporting information: (1) spirits (including premix); (2) beer; (3) cider; and (4) wine. In Australia, alcoholic ginger beer with alcohol by volume (ABV) more than or equal to 8% are taxed similarly to cider, whereas alcoholic ginger beer with ABV less than 8% are taxed similarly to beer. Accordingly, alcoholic ginger beer more than or equal to 8% was categorised with cider and less than 8% with beer [22]. Premixes were combined with spirits for analysis because of the similarity in the taxation of these two product types. Wine was further classified into two categories based on vessel size, because the specific MUP threshold in the NT was designed to impact the price of low‐cost wine, which are usually products sold in larger vessel sizes. The categories were: vessel sizes less than 1 L and vessel sizes more than or equal to 1 L.

#### Nominal price per standard drink

Nominal price per standard drink, the outcome variable for aims 1 and 2, was calculated as:

$$\frac{\text{Listed price}}{\left(\text{Number of standard drinks}\times \text{Number of units}\;\mathrm{per}\;\text{pack for listed price}\right)},$$

with an Australian standard drink having 10 g of pure alcohol.

#### Real price per standard drink

The outcome variable for aim 3, the real price per standard drink, was derived from the nominal price per standard drink using the monthly consumer price index (CPI), Australia's measure of inflation [23]:

$$\frac{\text{Nominal price}\;\mathrm{per}\;\text{standard drink}\;}{{\mathrm{CPI}}_{\text{month}}/{\mathrm{CPI}}_{\mathrm{Oct}\;2024}}.$$

We used the overall CPI to account for the affordability of alcohol in comparison to that of overall living affordability in Australia.

### Data exclusion criteria

Only alcoholic beverages available for sale in‐store were included (e.g. zero‐alcohol drinks, products that were out of stock or those only available via delivery were excluded). Data with no price per standard drink were also excluded, as were outlier prices outside of cut‐off thresholds (Table S1 for outlier cut‐off thresholds), and outlier prices at the upper or lower end of the distribution in nominal price per standard drink that were more than or less than three times that of the next highest or lowest price, respectively, for each product. These cut‐offs were determined based on inspection of the outlier price data for values that were implausible (e.g. based on breaks in the distribution of observed prices for the individual products). Where there were multiple price observations for the same product (identified by the product identifier defined above) at the same time point, only the lowest unique nominal price per standard drink was included to obtain one price observation per product at each time point. Data excluded from analysis are outlined in the flow chart in Figure S1.

### Analysis

Aim 1 was addressed by examining the percentage of products in March 2025 that were under the previous MUP threshold of $1.30 per standard drink. We also identified whether the subset of products under $1.30 in March 2025 were sold across all of the included retailer stores. Aim 2 was addressed by determining whether any products priced under $1.30 in March 2025 were previously identified in the market in the preceding study months (October 2024 through February 2025).

To address aim 3, we used quantile regression [24], which estimates effects across different quantiles of the price distribution, therefore enabling examination of different prices at the more extreme ends of the price distribution, rather than only the mean price, as in ordinary least squares regression. Fixed‐effects panel quantile regression (performed using the *rqpd 0.6* package in R [25]) was undertaken to test for any significant differences in price between March 2025 and February 2025, and between March 2025 and the earlier study months (October 2024 to January 2025) at the 10th, 20th, 30th, 40th, 50th (median), 60th, 70th, 80th and 90th price percentiles, with the product identifier defining the panel of products over time. The month of February 2025 was analysed separately from earlier months as this was when the excise tax that applies to spirits, premix and beer was indexed (with indexation based on the upward movement of the CPI at a rate of 1.009) [23]. A *post hoc* sensitivity analysis was performed comparing February 2025 with the earlier months to facilitate comparison of price changes immediately after excise duty indexation with changes immediately after MUP repeal. Bootstrap estimates were computed with 10 000 replicates. Bonferroni‐corrected *P*‐values and 95% CI for the three pairwise comparisons were computed from the bootstrap estimates using the *emmeans 2.0.0* package in R [26]. Estimates with *P*‐value <0.05 were deemed to be statistically significantly different from 0.

The analyses were not pre‐registered and, therefore, should be considered exploratory.

## RESULTS

The flowchart (Figure S1) shows the number of price observations identified in the collected data and the number of price observations included in the final sample for analysis. There were 56 878 product price observations across the three retailers and all time points (including duplicates and pack size variants). Of these, 55 281 observations were for alcoholic beverages, with 25 652 observations for alcoholic beverages available for in‐store purchase. Price observations were excluded where: no price data were available (*n* = 842); price per standard drink could not be derived as the product information did not contain data on the alcohol volume, vessel size or pack size (*n* = 51); there were duplicate price per standard drink observations (*n* = 622; which occurred if the same product was listed more than once on the website); and price observations were invalid or outliers (*n* = 66; typically from incorrectly specified vessel size or pack size). At this stage, where the price observations for the same product at the same time point differed in price, the higher price(s) per standard drink were excluded (*n* = 2002). The final sample included 22 069 product price observations.

Table S2 shows the number of available products from each selected store (total observations: retailer 1: 8374; retailer 2: 1877; retailer 3: 11 818) and Table S3 shows the median, 10th and 90th price percentiles over time by retailer. The median and 90th price percentiles for retailer 2 were lower than those for retailer 1 and 3 because retailer 2 had fewer premium products, whereas the 10th percentile prices were relatively similar across the three retailers.

### Aim 1: Proportion of products in March 2025 after the repeal priced under the previous $1.30 per standard drink threshold

**FIGURE 1:**
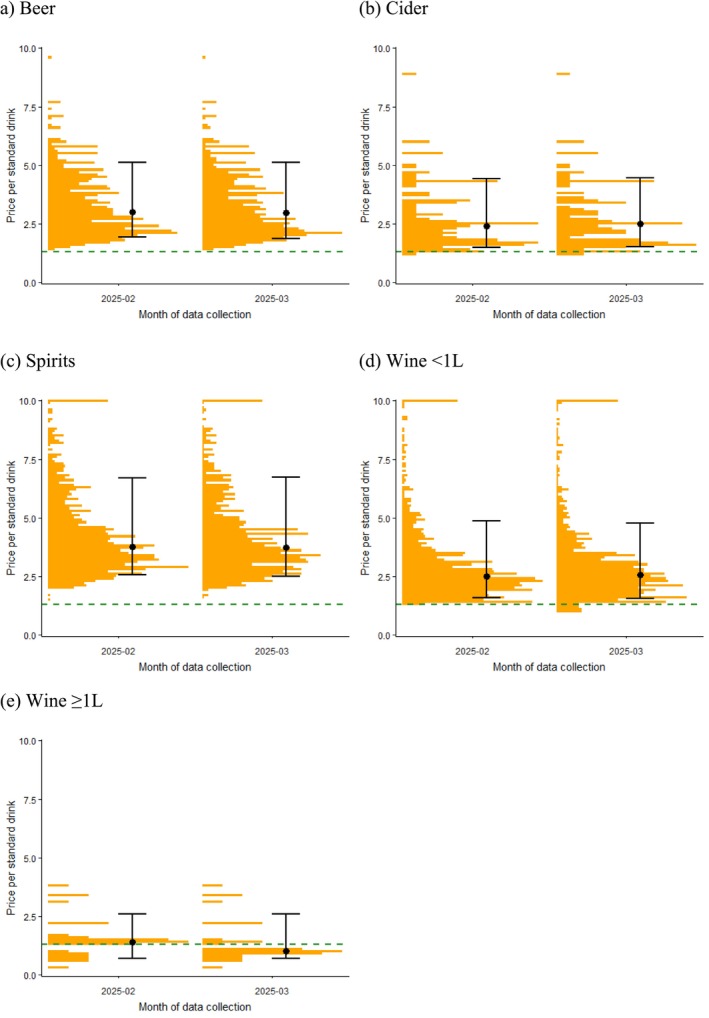
Distribution in nominal price per standard drink between February 2025 and March 2025. (a) beer, (b) cider, (c) spirits, (d) wine <1 L, (e) wine ≥1 L. The horizontal orange bars show the distribution in nominal price per standard drink in $0.10 increments. Prices over $10 per standard drink were grouped into the bar for $10 per standard drink for visibility. The vertical error bars show the range of the 10th and 90th percentiles of nominal price per standard drink. The round dot indicates the median price per standard drink. Green dashed horizontal line indicates where price per standard drink is $1.30. Plots including earlier months (October 2024 to January 2025) are presented in Figure S2.

The proportion of products priced under $1.30 a standard drink in March 2025 is shown in Table 1. In March 2025, there were no spirits (including premix) or beer priced under $1.30 per standard drink, and 2.5% of cider (3 products) and 5.1% of wine (78 products) were priced under $1.30 per standard drink. By vessel size, 3.5% of all wine in vessel sizes less than 1 L and 67.6% of all wine in vessel sizes 1 L or more were priced under $1.30 per standard drink (Figure 1). All 81 of these products were unique across the included retailer stores (however, at least 3 of them were available through 1 of the other 2 retailer stores for ≥$1.30).

**TABLE 1:** Proportion (*n*) of products priced below $1.30 per standard drink from October 2024 to March 2025.

Product type, % (*n*/*N*)	Before MUP repeal					After MUP repeal
	Oct 2024	Nov 2024	Dec 2024	Jan 2025	Feb 2025	Mar 2025
Spirits	0.0 (0/1289)	0.0 (0/1358)	0.0 (0/1329)	0.0 (0/1339)	0.0 (0/1347)	0.0 (0/1375)
Beer	0.0 (0/703)	0.0 (0/738)	0.0 (0/581)	0.0 (0/758)	0.0 (0/792)	0.0 (0/836)
Cider	7.7 (8/104)	7.3 (8/110)	4.7 (4/86)	3.2 (4/124)	1.6 (2/123)	2.5 (3/119)
Wine	0.7 (10/1484)	0.9 (13/1478)	0.7 (11/1502)	0.7 (10/1430)	0.7 (11/1528)	5.1 (78/1536)
<1 L	0.2 (3/1455)	0.1 (2/1443)	0.0 (0/1466)	0.0 (0/1395)	0.0 (0/1491)	3.5 (53/1499)
≥1 L	24.1 (7/29)	31.4 (11/35)	30.6 (11/36)	28.6 (10/35)	29.7 (11/37)	67.6 (25/37)
All unique products	0.5 (18/3580)	0.6 (21/3684)	0.4 (15/3498)	0.4 (14/3651)	0.3 (13/3790)	2.1 (81/3866)

Abbreviations: Dec, December; Feb, February; Jan, January; Mar, March; MUP, minimum unit price; Nov, November; Oct, October.

### Aim 2: Proportion of products found to be priced under $1.30 in March 2025 post‐repeal that were not previously available in preceding months

There were no spirits or beer priced under $1.30 in March 2025, therefore, analyses for this aim were focused only on wine and cider. Of the 81 products priced less than $1.30 per standard drink in March 2025, only one (1.2% of products) was not observed in any of the previous study months, which was a wine product in vessel size 1 L or more. The remaining 80 products priced less than $1.30 in March 2025 were available in at least one of the time points between October 2024 and February 2025.

### Aim 3: Shift in overall price distribution of alcohol products after the repeal of MUP in March 2025, compared with earlier months

Table S4 shows the 10th, 50th (median) and 90th price percentiles per standard drink by product type over the three time points. Consistently, wine sold in vessel sizes more than or equal to 1L had the lowest 10th, 50th and 90th price percentiles across the three time points, followed by cider, wine in vessel sizes less than 1 L, beer and spirits (Table S4).

#### Difference between March 2025 (post‐excise indexation and immediately post‐repeal) and February 2025 (immediately post‐excise indexation and pre‐repeal)

**FIGURE 2:**
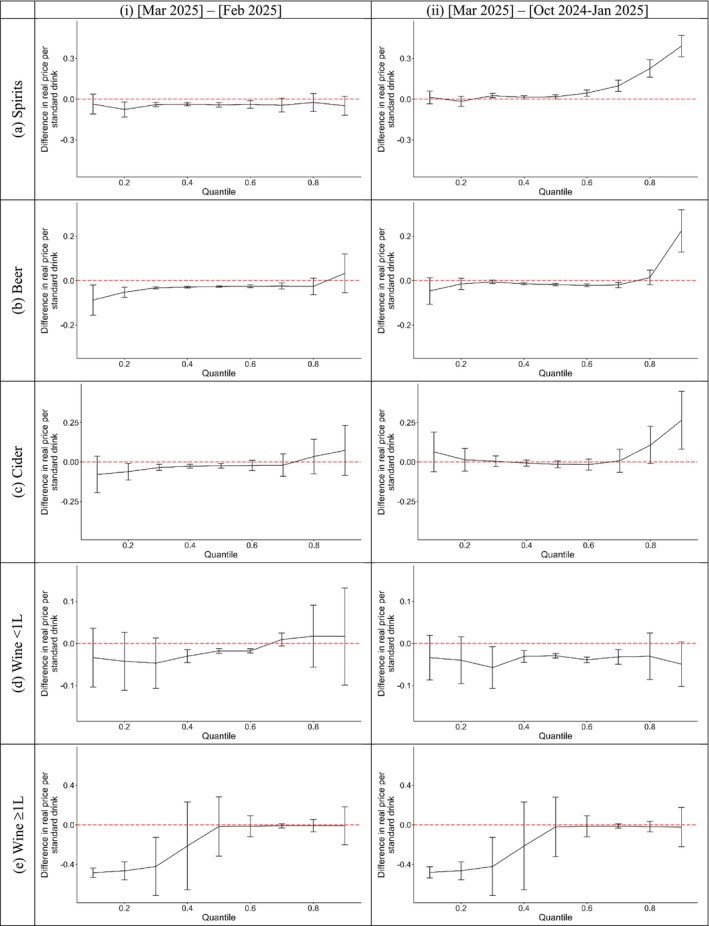
Difference in real price per standard drink in March 2025 (post‐excise indexation and immediately post‐repeal) compared with (i) February 2025 (immediately post‐excise indexation and pre‐repeal) and (ii) October 2024 to January 2025 (pre‐excise indexation and pre‐repeal). (a) spirits, (b) beer, (c) cider, (d) wine < 1 L, (e) wine ≥ 1 L. Real price was based on the price in October 2024 after adjusting for the monthly consumer price index. The vertical error bars show the 95% CI of difference in real price per standard drink at the included price percentiles, as indicated on the x‐axis. The intersection of the black line with the middle of the 95% CI is the estimated difference in real price per standard drink. The red dotted horizontal line indicates where there were no differences in real price per standard drink. Significant differences were indicated where the 95% CI does not cross the red dotted line.

The analysis of the change in price per standard drink across the price percentiles for March 2025 compared with February 2025 is presented in Table 2 and Figure 2(i). The price of spirits and beer, the two product types subject to the excise tax in February 2025, decreased across all percentiles between March and February 2025, with the exception of the most expensive beer which increased, although not significantly. The decreases for spirits and beer were significant around the middle of the price distribution (between the 20th and 60th percentiles for spirits and 10th and 70th percentiles for beer), with the largest significant decreases observed among cheaper products (−$0.08 per standard drink at the 20th percentile for spirits and −$0.09 per standard drink at the 10th percentile for beer).

**TABLE 2:** Estimated difference in price per standard drink by the 10th, 50th and 90th price percentiles.

	Mar 2025 versus Feb 2025		Mar 2025 versus Oct 2024–Jan 2025		Feb 2025 versus Oct 2024–Jan 2025	
	$0.00 (95% CI)	*P*‐value	$0.00 (95% CI)	*P*‐value	$0.00 (95% CI)	*P*‐value
Spirits						
90th price percentile	−0.05 (−0.12 to 0.02)	0.304	0.39 (0.31–0.47)	<0.001	0.44 (0.36–0.52)	<0.001
50th price percentile	−0.04 (−0.06 to −0.02)	<0.001	0.02 (0.00–0.03)	0.026	0.06 (0.04–0.08)	<0.001
10th price percentile	−0.04 (−0.11 to 0.04)	0.732	0.01 (−0.03 to 0.06)	>0.999	0.05 (−0.01 to 0.11)	0.135
Beer						
90th price percentile	0.03 (−0.05 to 0.12)	>0.999	0.22 (0.13 0.32)	<0.001	0.19 (0.09–0.29)	<0.001
50th price percentile	−0.03 (−0.03 to −0.02)	<0.001	−0.02 (−0.02 to −0.01)	<0.001	0.01 (0.00–0.01)	<0.001
10th price percentile	−0.09 (−0.16 to −0.02)	0.006	−0.05 (−0.11 to 0.01)	0.189	0.04 (−0.02 to 0.10)	0.247
Cider						
90th price percentile	0.07 (−0.08 to 0.23)	0.793	0.26 (0.08–0.45)	0.002	0.19 (0.05 to 0.33)	0.002
50th price percentile	−0.02 (−0.04 to −0.01)	<0.001	−0.01 (−0.04 to 0.01)	0.303	0.01 (−0.01 to 0.03)	0.806
10th price percentile	−0.08 (−0.19 to 0.04)	0.310	0.06 (−0.06 to 0.19)	0.658	0.14 (0.04–0.24)	0.002
Wine						
<1 L vessel						
90th price percentile	0.02 (−0.10 to 0.13)	>0.999	−0.05 (−0.10 to 0.00)	0.079	−0.07 (−0.17 to 0.04)	0.374
50th price percentile	−0.02 (−0.02 to −0.01)	<0.001	−0.03 (−0.03 to −0.02)	<0.001	−0.01 (−0.02 to −0.01)	<0.001
10th price percentile	−0.03 (−0.10 to 0.04)	0.743	−0.03 (−0.09 to 0.02)	0.390	−0.00 (−0.05 to 0.05)	>0.999
≥1 L vessel						
90th price percentile	−0.01 (−0.20 to 0.18)	>0.999	−0.02 (−0.22 to 0.18)	>0.999	−0.01 (−0.23 to 0.20)	>0.999
50th price percentile	−0.02 (−0.32 to 0.28)	>0.999	−0.02 (−0.32 to 0.28)	>0.999	−0.00 (−0.01 to −0.00)	0.041
10th price percentile	−0.48 (−0.53 to −0.44)	<0.001	−0.48 (−0.54 to −0.42)	<0.001	0.00 (−0.02 to 0.03)	>0.999

*Note*: Negative values indicate a reduction in price over time. The 90th price percentile represents the most expensive price segment, the 50th represents the median price and the 10th represents the least expensive price segment.

Abbreviations: Feb, February; Jan, January; Mar, March; Oct, October.

Cider and wine in vessel sizes less than 1 L both followed an overall similar pattern to beer between March and February 2025, however, the non‐significant increases in price were observed for cider at the 80th and 90th percentiles and for wine between the 70th and 90th percentiles, and the significant decreases were only observed between the 20th and 50th percentile for cider and the 40th and 60th for wine.

Across all product types, the greatest reductions in price between March and February 2025 were observed for wine in vessel sizes 1 L or more. Although there were decreases across the entire price distribution, the largest decreases were concentrated at the cheaper end of the market, with significant decreases of between −$0.42 per standard drink at the 30th percentile (95% CI = −0.72 to −0.13) and −$0.48 per standard drink at the 10th percentile (95% CI = −0.53 to −0.44).

#### Difference between March 2025 (post‐excise indexation and immediately post‐repeal) and October 2024 to January 2025 (pre‐excise indexation and pre‐repeal)

In contrast to the decreases observed for spirits across all percentiles between March and February 2025, the price of spirits predominantly increased between March 2025 and October 2024 to January 2025 [Figure 2(ii)]. All increases were statistically significant except those for the cheapest spirits, and the largest observed increase was for the most expensive spirits ($0.39 per standard drink; 95% CI = 0.31–0.47).

There were more consistent changes in the price of beer between March and February 2025 [Figure 2(i)(b)] and March 2025 and the earlier months [Figure 2(ii)(b)]. Decreases were observed between the 10th and 70th percentiles for the March 2025 and earlier months comparison, although significant decreases were only observed between the 40th and 70th percentiles and were of a smaller magnitude (−$0.01 at the 40th percentile and −$0.02 between the 50th and 70th percentiles). Unlike between March and February 2025, there was a significant increase of $0.22 per standard drink for the most expensive beer between March 2025 and the earlier months (95% CI = 0.13–0.32). A similarly large significant increase was seen between March 2025 and earlier months for the most expensive cider (90% percentile) ($0.26 per standard drink; 95% CI = 0.08–0.45). The pattern of changes for cider was otherwise inconsistent across the distribution with both small increases and small decreases and no significant findings at the other percentiles.

The price of wine in vessel sizes less than 1 L decreased across all price percentiles between March 2025 and the earlier months, with significant decreases between the 30th and 70th percentiles, and the largest decrease at the 30th percentile (−$0.06 per standard drink; 95% CI = −0.11 to −0.01). The pattern of findings for wine in vessel sizes 1 L or more between March 2025 and October 2024 to January 2025 was largely consistent with findings between March 2025 and February 2025, with decreases across the entire price distribution and of a similar magnitude, and the largest decreases at the cheaper end of the market (between the 10th and 30th percentiles).

#### *Post hoc* analysis of difference in pricing between February 2025 and October 2024 to January 2025

The estimates for the *post hoc* analysis are in Figure S3. For spirits, increases were seen across the price distribution between February 2025 and the earlier study months, and these were all of a greater magnitude than those observed between March 2025 and the earlier months. Across both analyses, the largest increases were for the most expensive spirits, at $0.44 per standard drink for February 2025 (95% CI = 0.36–0.52), compared to $0.39 for March 2025.

Although the prices for beer primarily decreased between March 2025 and the earlier months, prices primarily increased or remained stable for all price percentiles between February 2025 and the earlier months, with significant increases between the 20th and 50th percentiles, and at the 80th and 90th percentiles.

Although the increases in the price of cider at the more expensive end of the market (80th and 90th percentiles) were of a smaller magnitude between February 2025 and the earlier months than for March 2025 and the earlier months, the increases at the cheaper end of the market (between the 10th and 30th percentiles) were of a greater magnitude between February 2025 and the earlier months, with the largest increase at the 10th percentile ($0.14 per standard drink; 95% CI = 0.04–0.24).

For wine in vessel sizes less than 1 L, the decreases in price between February 2025 and the earlier months were of a smaller magnitude than those observed between March 2025 and the earlier months. Between February 2025 and the earlier months, the only significant decreases were between the 50th and 70th percentiles. Price patterns differed markedly between February 2025 and the earlier months and March 2025 and the earlier study months for wine in vessel sizes 1 L or more. Overall, prices remained stable or decreased by only $0.01 between February 2025 and the earlier months, compared to the large decreases between the 10th and 30th percentiles seen between March 2025 and the earlier months.

## DISCUSSION

To our knowledge, this study is the first to examine the impact of repealing MUP on alcohol prices, as illustrated in the NT, Australia. Within the first month of the repeal of the NT MUP, 78 wine products and three cider products in our sample were priced below the previous MUP threshold of $1.30 per standard drink. Although these products represented a small proportion of all alcohol products in our sample, they accounted for approximately two‐thirds of wine products sold in large vessel sizes, which were the high‐strength products the MUP was specifically designed to target, as well as 53 wine products in smaller vessel sizes. As such, our results demonstrate that the repeal of the MUP resulted in a rapid reduction in the prices of existing alcohol products in the NT market, increasing in particular the number of wine products available at cheaper price points.

Most products priced at or below $1.30 per standard drink in the month following the repeal had already been available in the market in previous study months, suggesting there was no immediate introduction of new or previously destocked products in response to the repeal. Although not one of our study aims, we did observe some wine and cider products in our sample that were priced below $1.30 while the MUP was in place, suggesting possible compliance issues. During the study period, the largest proportion of products priced below $1.30 was 0.6% in November 2024, when eight ciders, two wines in small vessel sizes and 11 wines in large vessel sizes were less than $1.30 per standard drink. Given our study commenced after the NT election in August 2024, we cannot determine whether these products were priced under $1.30 consistently while MUP was in place or if alcohol companies reduced the cost of these products in anticipation of the repeal. However, data from an official evaluation of the policy commissioned by the NT government showed that approximately 1% of all products in the year post implementation of MUP were non‐compliant [6]. Overall, although our sample shows that few new, cheap wine products were introduced in March 2025 following the MUP repeal, ongoing monitoring of product range and price will be crucial, particularly as industry responses such as the introduction of new products, reformulation of ABV or changes to pack and container sizes, would likely be implemented over a longer time period. This is particularly salient given an ancillary public health benefit of MUP is that it can limit the introduction of new, low‐cost products into the market because it caps the extent of price‐based competition possible [27]. Therefore, the repeal of the MUP in the Northern Territory will once again enable alcohol companies to down compete on price.

Notable shifts were observed in the overall price distribution of alcohol in our sample in March 2025, particularly compared to the month pre‐repeal. With the exception of the most expensive spirits and beer products (those at the 90th price percentile), all other spirits and beer products increased in price in February 2025 when indexation of the volumetric excise tax occurred. However, unlike in other jurisdictions such as New South Wales (NSW), where we have seen sustained price increases for these product types both in the month of excise indexation and the month following [28], the price of spirits and beer in the Northern Territory decreased in March 2025 after the repeal. This reduction in prices for two product types well above the MUP threshold of $1.30 per standard drink suggests that the MUP repeal may have exerted downward pressure on the price of alcohol across the whole market, not just the cheapest products. As such, it appears that the repeal of the MUP may have to some extent reversed the price increases that occurred across the market when the policy was implemented [6].

Substantial changes were also observed in the price distribution of wine and cider. The cheapest one‐third of wine products in large vessel sizes decreased significantly in price in March 2025 compared to February 2025. However, we found that these declines occurred against a backdrop of longer‐term price decreases in wine before the MUP repeal, which aligns with our previous findings in NSW that products taxed under the Wine Equalisation Tax (WET)—an ad valorem (value‐based tax) applied to wine and cider products—either decreased or remained stable in price over time [29]. A more unexpected pattern emerged for cider, whereby the 10th, median and 90th percentile prices were higher in March 2025 compared to October 2024 to January 2025. There were on average more cider products priced below $1.30 in October 2024 to January 2025 than there were in March 2025, despite the MUP being in place, which may in part explain this result, particularly given the relatively small size of the cider market compared with other product types. Overall, although our results show that the MUP was generally effective in controlling the price of alcohol at the cheaper end of the market, the median prices of cider and wine in large vessel sizes were substantially lower than for other product types even when the policy was in effect. The implementation of a volumetric tax for all alcohol products would likely work to reduce the current price disparities between different product types.

### Limitations

There are several limitations of our study to consider. First, our data were drawn exclusively from retailers in Darwin, the NT's capital city. Anecdotally, alcohol was already priced above $1.30 per standard drink in remote areas of the Northern Territory when the MUP was implemented due to high freight costs [30]. Consequently, the impact of the implementation, and now the repeal, of MUP on alcohol price may have varied substantially between the regional centre of Darwin and more geographically dispersed areas. Research in remote areas is needed, particularly as the majority of individuals living in these locations are Aboriginal and Torres Strait Islander people who experience disproportionate levels of alcohol‐related harm [21]. Additionally, although we included all products available at three of the largest alcohol retailers in Australia, there may be variations in both product range and price in smaller or independent retailers that were not captured in our study.

The unique setting and geography of the Northern Territory should be considered when determining the generalisability of our findings to other jurisdictions. A matched case–control study comparing the same products in other Australian jurisdictions could help determine the extent to which our findings in the Northern Territory may reflect local conditions as compared to broader policy effects. We were also unable to control for seasonality because of the short timeframe of our study, and therefore, cannot determine whether any changes in price were influenced by the increased demand for alcohol typically observed in warmer seasons [31]. The 95% CIs were computed as asymptotic estimates, and it is likely that the CIs were asymmetrical around the estimated differences in the quantile regression model. We note, however, that statistical significance as determined by *P* < 0.05 was generally consistent with the 95% CIs.

## CONCLUSION

Overall, our study has shown an immediate increase in the number of alcoholic products available under $1.30 in Darwin, Northern Territory in the first month after the repeal of the MUP, and that these were predominantly wine products. Longer‐term price monitoring is required to measure any further shifts in alcohol price, as well as any changes in market composition (e.g. the introduction of new products) that may correspond to the MUP repeal, and wholesale data, alongside data on alcohol harms, should be examined to determine any corresponding shifts in consumption and harms. Monitoring pricing strategies used by the alcohol industry such as promotions and volume discounts will also be important, given that the MUP was the only policy restricting the capacity of retailers to out‐compete through price.

## AUTHOR CONTRIBUTIONS

**Mia Miller:** Conceptualization (equal); methodology (equal); project administration (equal); writing—original draft (lead); writing—review and editing (lead). **Nicola Man:** Conceptualization (equal); data curation (lead); formal analysis (lead); methodology (equal); writing—original draft (supporting); writing—review and editing (equal). **Nicholas Taylor:** Conceptualization (equal); methodology (equal); writing—review and editing (equal). **Michael Livingston:** Conceptualization (equal); methodology (equal); writing—review and editing (equal). **Sarah Clifford:** Conceptualization (equal); writing—review and editing (equal). **Michala Kowalski:** Conceptualization (equal); methodology (equal); writing—review and editing (equal). **Heng Jiang:** Conceptualization (supporting); writing—original draft (supporting); writing—review and editing (supporting). **Sarah Callinan:** Conceptualization (supporting); writing—original draft (supporting); writing—review and editing (equal). **Cassandra J. C. Wright:** Conceptualization (equal); writing—review and editing (equal). **Amy Peacock:** Conceptualization (equal); methodology (equal); project administration (equal); supervision (equal); writing—original draft (supporting); writing—review and editing (equal).

## DECLARATION OF INTERESTS

A.P. has received untied educational grants from Seqirus and Mundipharma for post‐marketing surveillance of pharmaceutical opioids. All other authors have no conflicts of interest to declare.

## Supporting information


**Data S1.** Supporting Information.

## Data Availability

The data that support the findings of this study are available from the corresponding author upon reasonable request.
