# Supplementary material for: Evaluating the immediate impacts of the repeal of the Northern Territory minimum unit price on off‐premises alcohol pricing: Findings from an observational study of online alcohol retailers
Source: Addiction. 2026 Jun 16;121(9):2362–72. doi: 10.1111/add.70466 (PMC13486911; doi:10.1111/add.70466)
Supplement: Supplementary file 1 [file ADD-121-2362-s001.pdf]

Supplementary material

Algorithm for categorisation of alcoholic beverages into product types

The following outlines the approach in the algorithm for the categorisation of the product types:

1) The product type variable was initalised using the menu bar data on the website.
2) The product type variable was redefined using the product specification data (e.g., type, style and varietal) to further identify a product type. This was determined firstly by cross-tabulating the product specification data with the menu bar data from step (1) to define product specification terms that identify distinct product types, then determine whether product specification terms that cross-classify into >1 product type can be classified into a single product type, e.g., “vermouth” was identified both as spirits and wine from the menu bar data on product type but it is categorised as a wine in this step. Ambiguous terms were also excluded, e.g., "other drinks",”non-alc”, “yes”. The procured terms for each product type were then used to redefine the product type variable.
3) Keywords were identified in the product name or brand to differentiate and redefine a product type such as:
    a. product names that identify a product type, e.g., “Martini” to differentiate a spirit product as a premix, “vermouth” to identify (fortified) wine.
    b. flavour descriptors, e.g., “Cola”, “passionfruit” to identify flavoured spirits as premixes.
    c. specific brands that primarily produce a particular product type, e.g., a “Vodka Cruiser” product as a premix.
4) Ginger beer were further identified using specific keywords and product type (e.g., “ginger” in product name under beer or cider as a product type, but not if it was under wine). This step was carried out so that it could be categorised based on ABV into beer (ABV $< 8\%$) and cider (ABV $\geq 8\%$)
5) The whole dataset was finally checked for consistency of product type categorisation between listings of the same product. A product may occasionally be identified as a different product type in separate listings because the menu bar and product specification data may differ for separate listings of the same product. This generally occurred for the two scenarios below:
    a. A product was identified as spirits and premix in separate listings: the product was categorised as a premix in this scenario.
    a. A product was identified as beer and cider in separate listings: the product was categorised as a cider in this scenario.

Steps (2) and (3) were needed to differentiate premixes from spirits, and between cider, ginger beer and beer because the menu bar data did not differentiate these product types for some of the retailers. These two steps also addressed misclassified data or products that were

not consistently classified into a particular product type, e.g., seltzer was occasionally classified as beer because some breweries produced seltzers, and vermouth as a fortified wine was occasionally classified as a spirit. Step (2) and (3) involved iterating through a set of criteria in order of priority, i.e., criteria that more clearly differentiate a product type took precedence over more ambiguous criteria. Even though premix were included within spirits as a product type in this study, it was separated out to identify outlier prices based on the cutoff thresholds in Table A.

Keywords in step (3) were procured through the examination of listings that were likely to be misclassified, e.g., listings with outlier prices, ABVs or vessel size within the product type. Step (3) may additionally utilise ABV and vessel size to further differentiate certain product types, e.g., defining an ambiguous spirit product as a premix if it was in a small vessel, i.e., vessel size <400mL and ABV <10% (and not identified as a zero-alcohol product), or if a spirit product had "Vodka", "Whisky", "Whiskey", "orange", etc. in the product name and ABV <10% (and not identified as a zero-alcohol product).

The reclassified and outlier product listings were examined during the development of the algorithm to check the performance of the classification criteria, e.g., the term "cider" in the product name was capturing a gin product and exclusion of listings with the term "gin" in the product name was added to this criterion for defining cider as a product type. Note that a small proportion of products may still be misclassified after the application of the classification algorithm.

Table S1. Cutoff thresholds for outlier price observations

	Product type				
	Spirits	Cider	Premix	Beer	Wine
Price per unit					
Lower threshold	$0.25	$0.25	$0.25	$0.25	$0.25
Upper threshold	--	--	--	--	--
Price_100mL					
Lower threshold	$0.20	$0.20	$0.20	$0.20	$0.20
Upper threshold	$300.00	$4.00	$10.00	$4.00	$100.00
Price per standard drink					
Lower threshold	$0.60	$0.20	$0.60	$0.60	$0.20
Upper threshold	--	$10.00	$10.00	$10.00	--

Note: Premixes were included within the spirits category for the analyses in this study.

Table S2. Percentage (and number) of product observations by store

Retailer	Oct 2024	Nov 2024	Dec 2024	Jan 2025	Feb 2025	Mar 2025	Total
1	36.2 (1296)	38.7 (1425)	34.7 (1213)	37.8 (1381)	40.1 (1519)	39.8 (1540)	37.9 (8374)
2	8.3 (298)	8.4 (310)	8.9 (312)	8.5 (311)	8.5 (321)	8.4 (325)	8.5 (1877)
3	55.5 (1986)	52.9 (1949)	56.4 (1973)	53.7 (1959)	51.5 (1950)	51.8 (2001)	53.6 (11818)
Total	100.0 (3580)	100.0 (3684)	100.0 (3498)	100.0 (3651)	100.0 (3790)	100.0 (3866)	100.0 (22069)

Note: The selected store for the retailer 2 closed between the December 2024 and January 2025 data collection; an alternative store from this retailer was selected for data collection from January 2025 until March 2025.

Table S3. Median price per standard drink (with 10th and 90th percentiles) by store

Retailer	Oct 2024	Nov 2024	Dec 2024	Jan 2025	Feb 2025	Mar 2025
1	3.17 (1.77, 5.75)	3.23 (1.82, 5.91)	3.21 (1.82, 6.26)	3.24 (1.84, 5.99)	3.22 (1.84, 5.79)	3.23 (1.88, 5.99)
2	2.67 (1.91, 4.33)	2.65 (1.88, 4.32)	2.64 (1.87, 4.49)	2.64 (1.84, 4.51)	2.79 (1.91, 4.56)	2.65 (1.76, 4.45)
3	2.99 (1.69, 5.49)	2.95 (1.69, 5.51)	2.94 (1.63, 5.49)	3.04 (1.75, 5.54)	2.95 (1.69, 5.76)	3.03 (1.63, 5.79)

Figure S1. Data exclusion flowchart

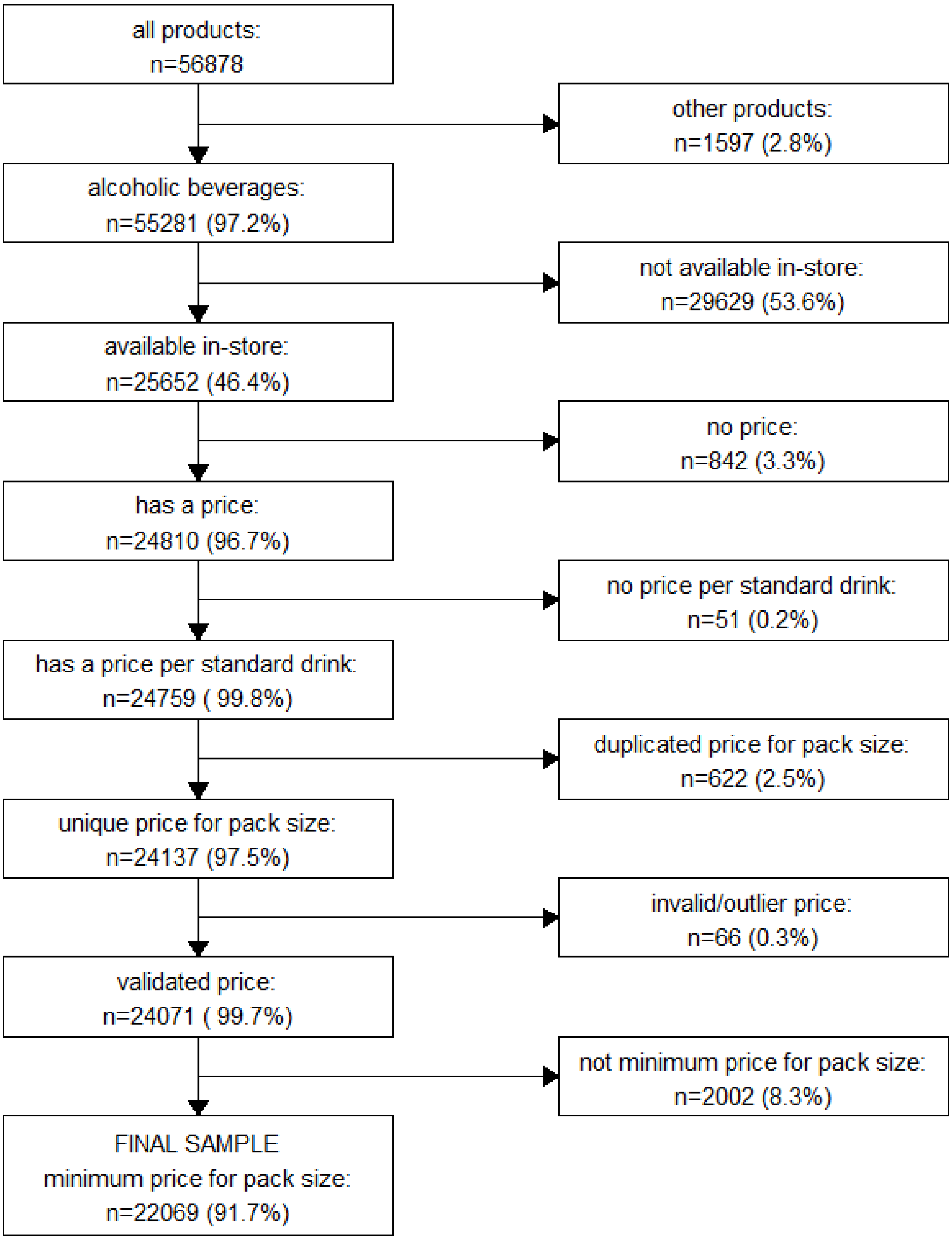

Note: n=number of price field observations

Figure S2. Distribution in nominal price per standard drink over time for by product type.

(i) Spirits

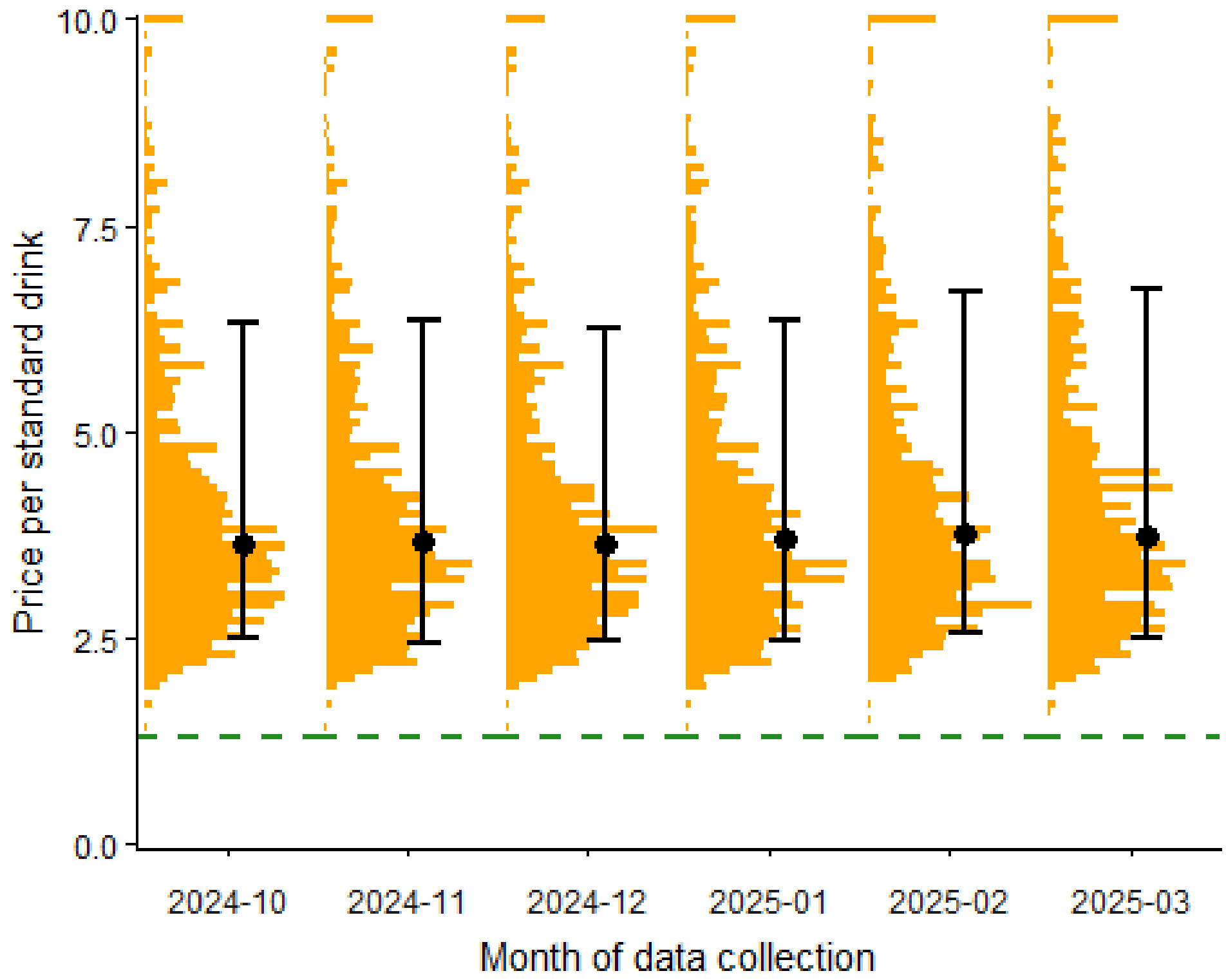

(ii) Beer

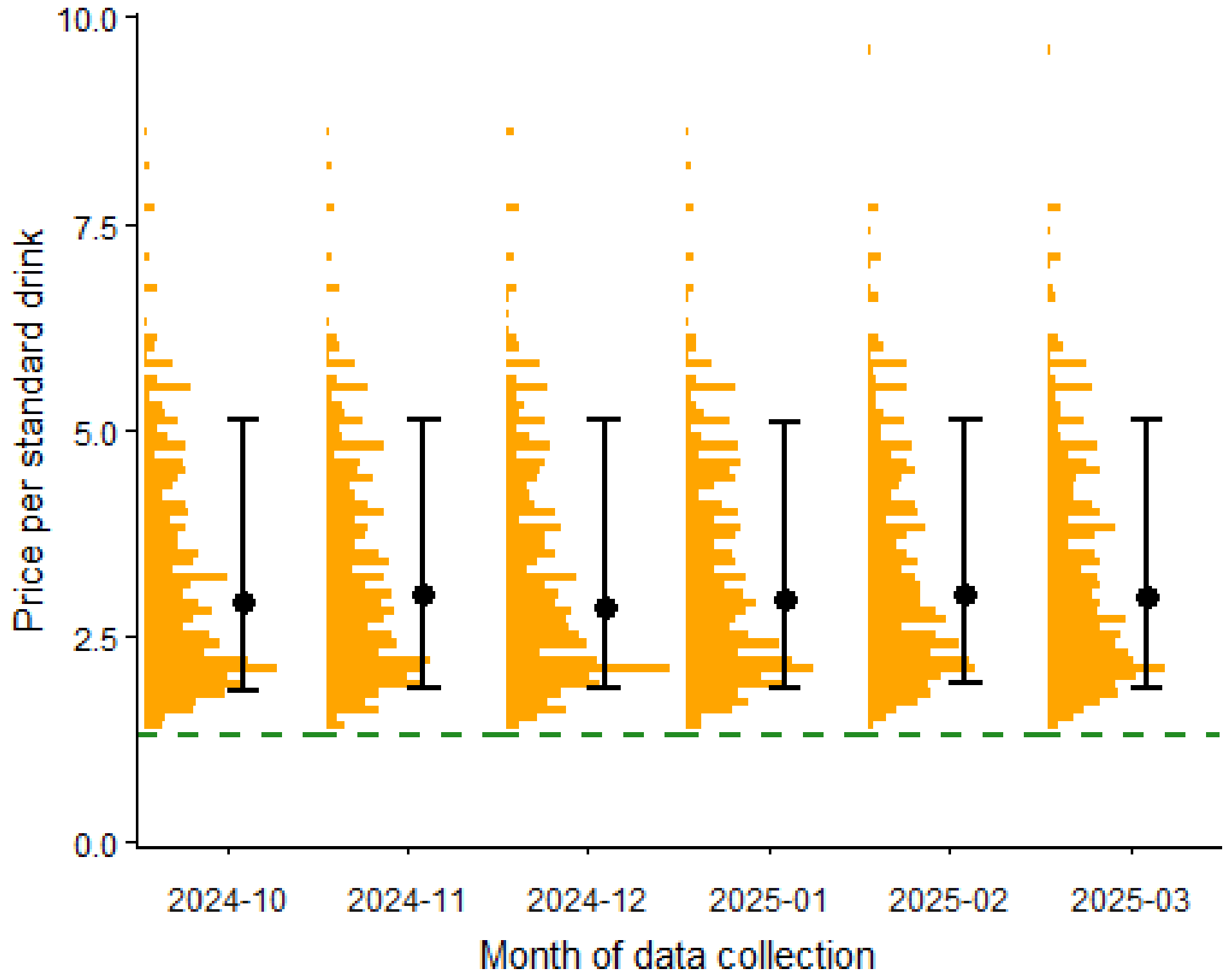

(iii) Cider

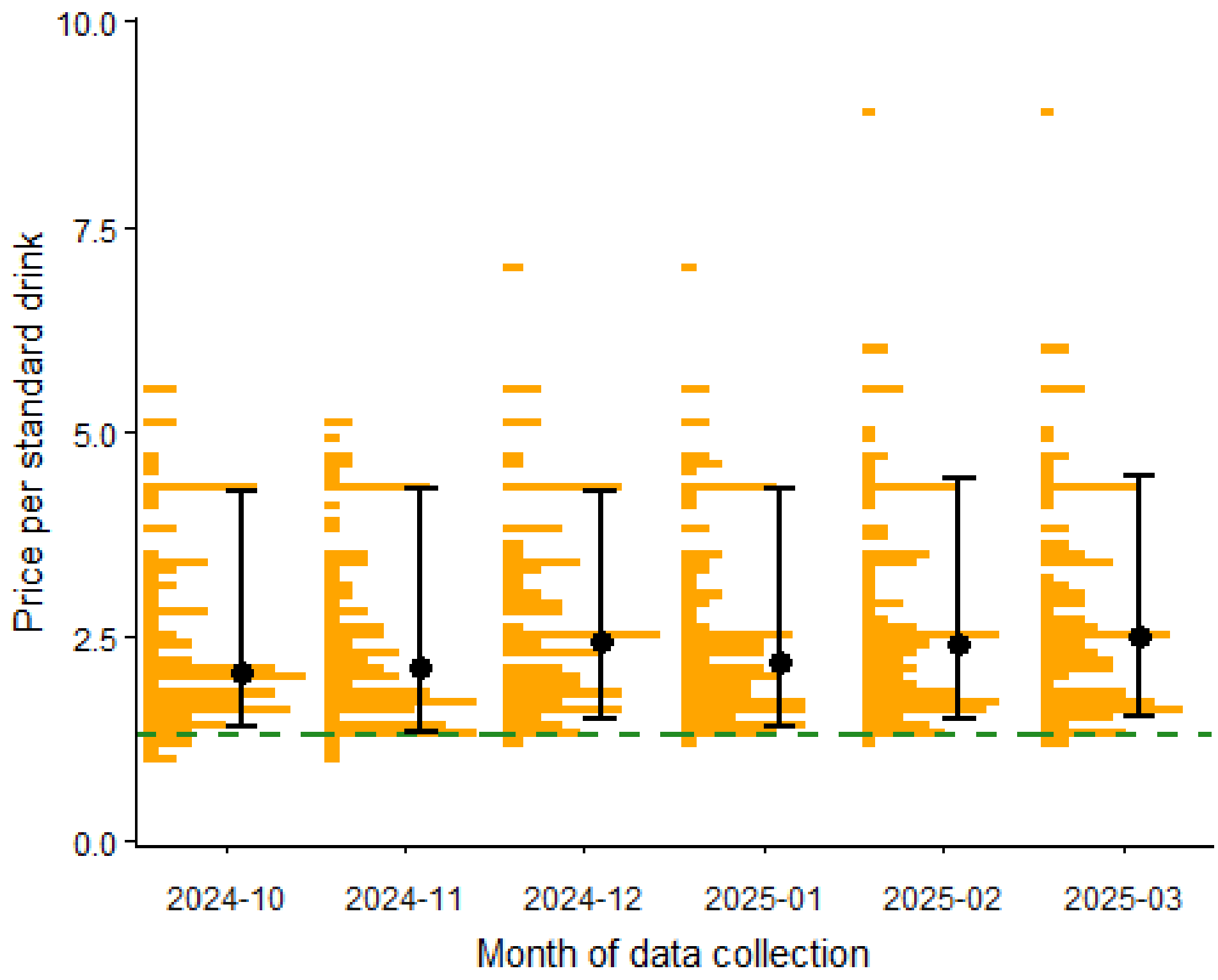

(iv) Wine

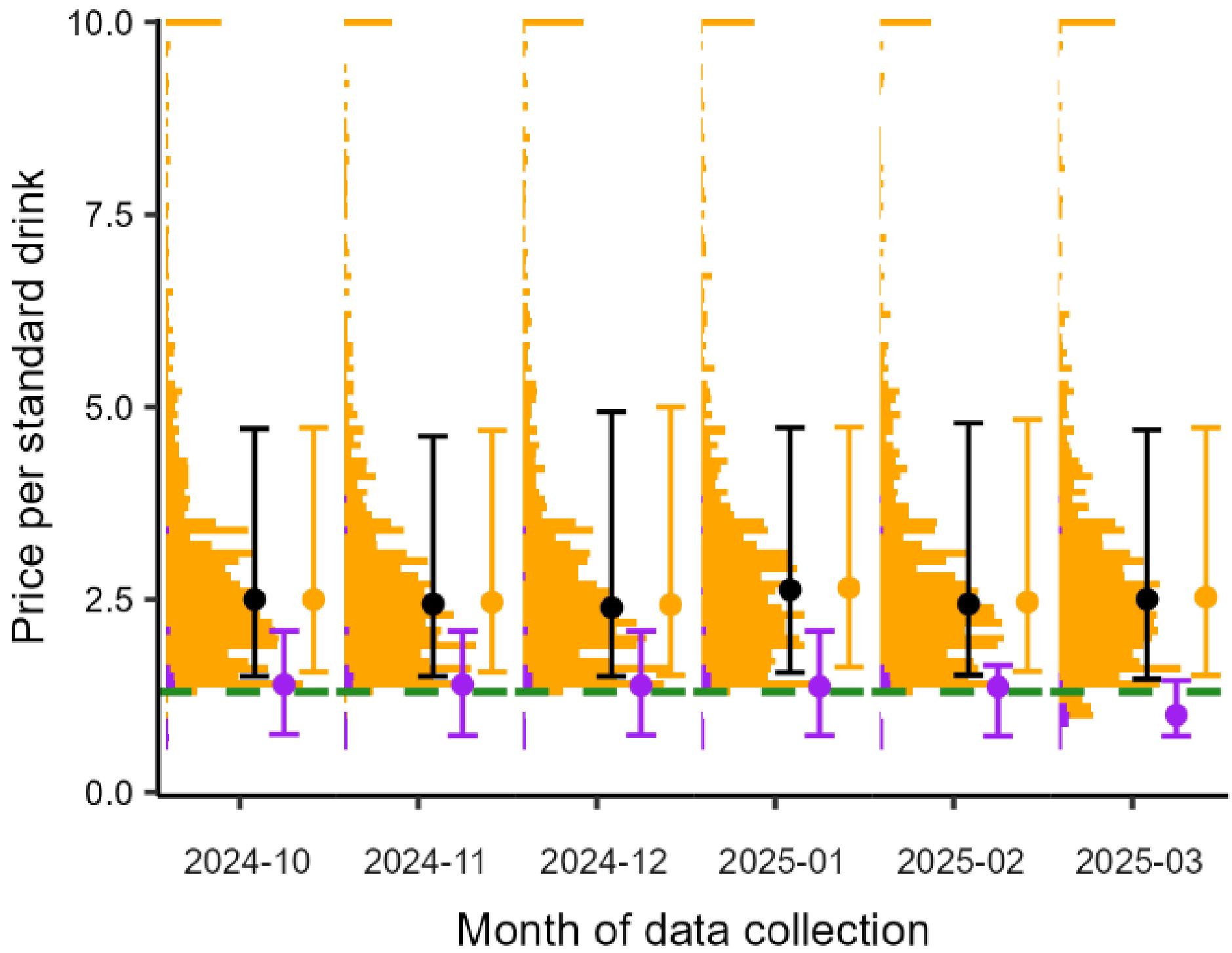

Note: The horizontal orange bars show the distribution in nominal price per standard drink in $0.10 increments for each product type in (i) to (iii) and for wine <1L in (iv). The horizontal purple bars show the distribution in nominal price per standard drink in $0.10 increments in

for wine ≥1L in (iv). Prices over $10 per standard drink were grouped into the bar for $10 per standard drink for visibility. The black vertical error bars show the range of the 10th and 90th percentiles of nominal price per standard drink for each product type. The black round dot indicates the median price per standard drink. The orange vertical error bar and dot are the 10th and 90th percentile and median for wine <1L in size and the purple vertical error bar and dot are the 10th and 90th percentile and median for wine ≥1L in size. Green dashed horizontal line indicates where price per standard drink is $1.30.

Table S4. Median (10th and 90th percentiles) real price per standard drink by product type over time

Product type	Oct 2024-Jan 2025	Feb 2025	Mar 2025
**Spirits**	3.63 (2.44, 6.27)	3.72 (2.53, 6.61)	3.68 (2.46, 6.61)
**Beer**	2.91 (1.85, 5.08)	2.98 (1.89, 5.07)	2.93 (1.84, 5.04)
**Cider**	2.14 (1.37, 4.26)	2.36 (1.45, 4.36)	2.45 (1.48, 4.38)
**Wine**	2.50 (1.50, 4.73)	2.44 (1.53, 4.74)	2.49 (1.47, 4.64)
<1L	2.53 (1.55, 4.80)	2.48 (1.56, 4.82)	2.52 (1.52, 4.69)
≥1L	1.39 (0.68, 2.33)	1.38 (0.70, 2.57)	1.00 (0.69, 2.55)

Note: Real price was based on the price in October 2024 after adjusting for the monthly consumer price index.

Figure S3. Estimated difference in real price per standard drink percentiles in February 2025 compared with October 2024 to January 2025

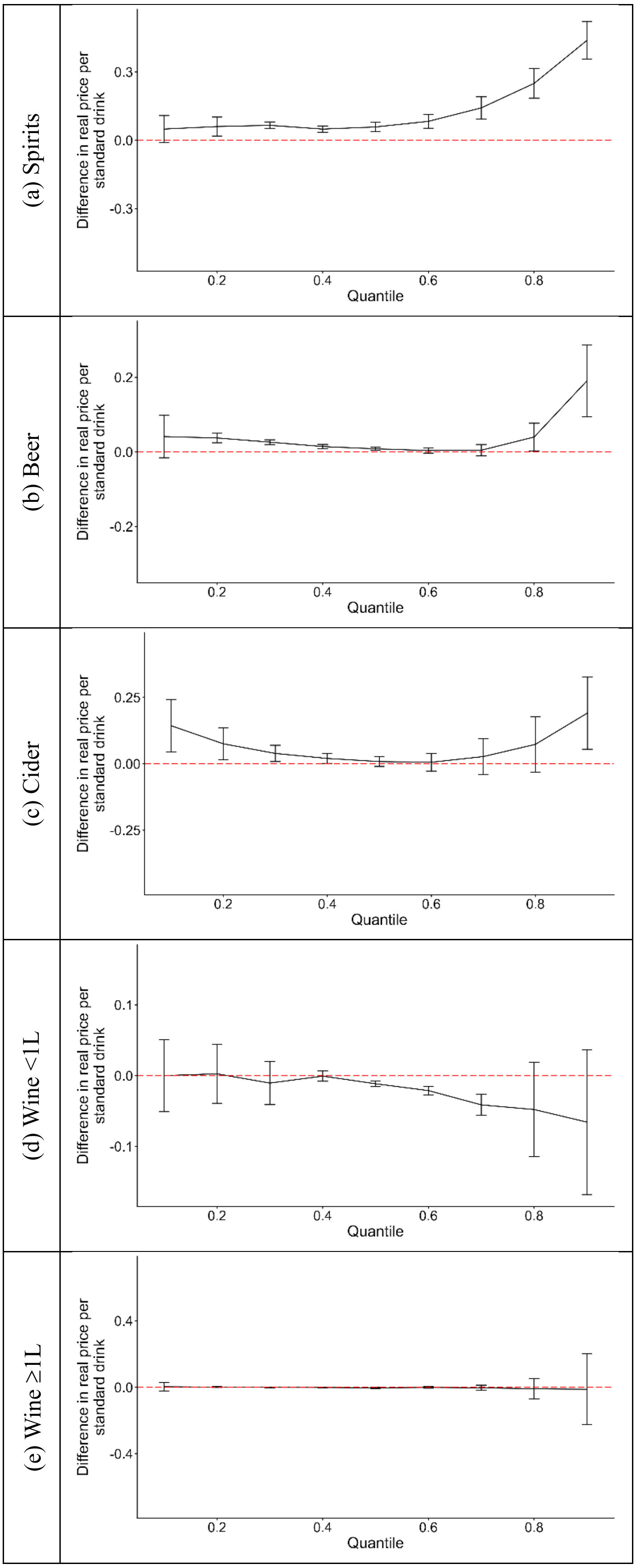

Note: Real price was based on the price in October 2024 after adjusting for the monthly consumer price index. The vertical error bars show the 95% confidence interval of difference in real price per standard drink at the included price percentiles as indicated on the x-axis. The intersection of the black line with the middle of the 95% confidence interval is the estimated difference in real price per standard drink. The red dotted horizontal line indicates where there were no difference in real price per standard drink. Significant differences were indicated where the 95% confidence interval does not cross the red dotted line.
